# Study of Peri-Articular Anaesthetic for Replacement of the Knee (SPAARK): study protocol for a patient-blinded, randomised controlled superiority trial of liposomal bupivacaine

**DOI:** 10.1186/s13063-019-3826-1

**Published:** 2019-12-16

**Authors:** Ruth Knight, Lisa Poulton, Louise H. Strickland, Thomas W. Hamilton, David Beard, Jonathan Cook, Susan J. Dutton, Jose Leal, Sarah Lamb, Cushla Cooper, Karen L. Barker, David W. Murray, Hemant G. Pandit

**Affiliations:** 1 Oxford Clinical Trials Research Unit, Centre for Statistics in Medicine, Nuffield Department of Orthopaedics, Rheumatology and Musculoskeletal Sciences, University of Oxford, Oxford, UK; 2 Surgical Interventional Trials Unit, Nuffield Department Orthopaedics, Rheumatology and Musculoskeletal Sciences, University of Oxford, Oxford, UK; 3 Oxford Orthopaedic Engineering Centre, Nuffield Department of Orthopaedics, Rheumatology and Musculoskeletal Sciences, University of Oxford, Oxford, UK; 4 Health Economics Research Centre, Nuffield Department of Population Health, University of Oxford, Oxford, UK; 5 National Institute for Health Research Biomedical Research Unit, Nuffield Department of Orthopaedics, Rheumatology and Musculoskeletal Sciences, University of Oxford, Oxford, UK; 6 Physiotherapy Research Unit, Nuffield Orthopaedic Centre, Oxford University Hospitals, NHS Foundation Trust, Oxford, UK; 7 Leeds Institute of Rheumatic and Musculoskeletal Medicine, University of Leeds, Leeds, UK

**Keywords:** Randomised controlled trial, Knee replacement, Liposomal bupivacaine, Anaesthetic, Analgesia, Peri-operative pain

## Abstract

**Background:**

Optimising the management of peri-operative pain and recovery following knee replacement has been identified as a patient priority. Current pain relief strategies use opiate-based analgesia; however, up to 50% of patients experience significant side effects. Local anaesthetic incisional infiltration is one alternative. The length of the duration of action is a major limiting factor of current local anaesthetic techniques. Liposomal bupivacaine has been reported to be effective for up to 72 h. This randomised controlled trial will evaluate the clinical and cost effectiveness of liposomal bupivacaine.

**Methods:**

SPAARK is a patient-blinded, multi-centre, active comparator, superiority, two-arm, parallel-group randomised controlled trial. Five hundred patients undergoing knee replacement will be recruited and randomised to liposomal bupivacaine plus bupivacaine hydrochloride or bupivacaine hydrochloride alone. The co-primary outcomes are the Quality of Recovery 40 measured at 72 h post-surgery and also cumulative pain measured daily using a 0–10 visual analogue scale for the first 3 days following surgery. Secondary outcomes include cumulative opioid consumption, fitness for discharge, functional outcomes assessed using the Oxford Knee Score and American Knee Society Score, the EuroQol five dimensions instrument and complications. A cost utility analysis is also planned.

**Discussion:**

The clinical effectiveness and cost effectiveness of liposomal bupivacaine have yet to be evaluated in the National Health Service, making this trial appropriate and timely.

**Trial registration:**

ISRCTN registry, ISRCTN54191675. Registered on 14 November 2017.

## Background

Around 100,000 primary knee replacements are performed in the UK every year, and this number is increasing. Similar trends are likely to be seen worldwide, with the number of primary total knee arthroplasties predicted to rise to 3.48 million procedures by 2030 [1]. Optimising the management of peri-operative pain and recovery has been identified as a patient priority [2]. Improving post-operative recovery has many benefits both to the patient in terms of reduced morbidity and mortality and the healthcare system through reduced healthcare-associated costs [3]. Current pain relief strategies post-knee replacement surgery use opiate-based analgesia. However, up to 50% of patients experience significant side effects, such as nausea, vomiting and drowsiness [4]. Opioid-sparing techniques have been associated with enhanced patient satisfaction post-surgery [5]. There is increasing evidence about their longer term benefits, with studies reporting a lowered incidence of chronic post-surgical pain, though the full effect on long-term patient-reported outcome measures is unknown [6–8].

The concept of multi-modal analgesia was introduced more than 20 years ago, and its use has expanded to many areas of surgery, including knee replacement surgery [9]. Local anaesthetic incisional infiltration is commonly used as part of this technique with the view that modification of pain stimuli at their origin will reduce the transmission of nociceptive stimuli. The length of their duration of action is a major limiting factor of current local anaesthetic techniques, and despite multi-modal techniques, a significant number of patients report severe post-operative pain following knee replacement. Current methods to address this remain unsatisfactory due to the increased risk of infection, interference with early mobilisation and discharge caused by the use of an indwelling infiltration catheter as well as the additional cost of infusion devices. Due to the inherent benefits of having a longer acting local anaesthetic, a great deal of interest has been invested in the development of a new drug: liposomal bupivacaine.

Liposomal bupivacaine (Exparel, Pacira Pharmaceuticals, Inc., San Diego, CA, USA) is liposome-encapsulated bupivacaine, which has been reported to be effective for up to 72 h [10, 11]. Using a proprietary technique known as DepoFoam (SkyePharma, London, UK), the local anaesthetic bupivacaine is held within multi-vesicular liposomes which permit prolonged release through diffusion, membrane breakdown and re-organisation [12].

Previous studies using liposomal bupivacaine show that some patients were discharged on the day of surgery following hip and knee replacement procedures with no reported incidents of increased re-admission rates or wound issues [13–15].

These studies also show that liposomal bupivacaine is well tolerated and has been reported to be associated with significantly lower pain scores, opiate usage and opiate-related adverse effects as well as a reduced length of stay. Liposomal bupivacaine has also been reported to be cost effective in gastrointestinal and orthopaedic joint surgery in the USA [13]. The clinical effectiveness and cost effectiveness of liposomal bupivacaine have yet to be evaluated in the National Health Service (NHS), making this trial appropriate and timely.

## Methods/design

### Trial design

The Study of Peri-Articular Anaesthetic for Replacement of the Knee (SPAARK) is a patient-blinded, multi-centre, active comparator, superiority, two-arm, parallel-group randomised controlled trial. The aim of SPAARK is to evaluate the clinical and cost-effectiveness of liposomal bupivacaine with bupivacaine hydrochloride compared to bupivacaine hydrochloride alone on post-operative recovery after knee replacement surgery. Eligible patients listed for knee replacement will be randomised to either liposomal bupivacaine plus bupivacaine hydrochloride or bupivacaine hydrochloride alone. Randomisation will be performed using a secure on-line Internet-based system (RRAMP) provided by the Oxford Clinical Trials Research Unit (OCTRU).

Patients will be randomised 1:1 to the two treatment groups using stratified randomisation with variable block sizes, stratified by recruitment centre and surgical procedure (total knee replacement (TKR) or unicompartmental knee replacement (UKR). The co-primary outcome measures for SPAARK are cumulative pain, measured daily using a 0–10 visual analogue scale (VAS) for the first 3 days post-surgery, summarised using the area under the curve, and Quality of Recovery 40 (QoR-40) score measured at 72 h. Further follow-up assessments with patient-reported questionnaires will be performed 6 weeks after surgery and 6 and 12 months after randomisation. These include the Oxford Knee Score (OKS), the patient-reported section of the American Knee Society Score (AKSS), a health resource use questionnaire and the EuroQol five levels of severity and five dimensions instrument (EQ-5D-5 L). Data on adverse events is also collected from sites and participants.

The protocol conforms to the Standard Protocol Items: Recommendations for Interventional Trials (SPIRIT) guidelines [16]. The SPIRIT checklist is provided as Additional file 1. The data collected at each time point will be as described in Table 1.

**Table 1:** Summary of outcomes and assessment schedule

Time point	Baseline	Operation	Day 0	Day 1	Day 2	Day 3	6 weeks	6 months	12 months
Informed consent	X								
Patient demographics	X								
Summary medical history	X								
Summary medication history	X						X	X	X
QoR-40	X		X	X	X	X	X	X	X
Pain VAS at rest	X		X	X	X	X	X	X	X
Cumulative opioid consumption			X	X	X	X			
Fitness for discharge			X	X	X	X			
Oxford Knee Score	X						X	X	X
American Knee Society Score	X						X	X	X
Patient expectations	X						X	X	X
EQ-5D-5 L	X		X	X	X	X	X	X	X
Summary healthcare/social services use and informal care due to knee	X						X	X	X
Summary employment history	X						X	X	X
Treatment		X							
Knee replacement procedure details		X							
Administration of investigational medicinal product		X							
Complications		X	X	X	X	X			

### Trial setting

Five hundred participants will be recruited from approximately ten sites across the UK. The number of recruiting centres may be increased as the study progresses. The recruiting centres are a mix of district general hospitals and specialist centres from a range of regions across England.

### Inclusion criteria

The participant may enter the trial if *all* of the following apply:

- Has had unilateral primary knee replacement, including both TKR and UKR, for end-stage osteoarthritis
- Has American Society of Anesthesiologists (ASA) physical status grades I to III
- Is willing and able to consent for himself/herself
- Is male or female, aged 18 years or older
- In the Investigator’s opinion, is able and willing to comply with all trial requirements.

### Exclusion criteria

The participant may not enter the trial if *any* of the following apply:

- Allergy or intolerance to amide-type local anaesthetics
- Objective evidence of nerve damage in the affected lower limb
- Rheumatoid arthritis
- Any other significant disease, disorder or condition which, in the opinion of the Investigator, may either put the participants at risk because of participation in the trial, or may influence the results of the trial or the participant’s ability to participate in the trial
- Participation in another research trial involving an investigational product in the past 6 months
- Patients who have significant cognitive impairment or language issues
- Contra-lateral knee replacement within the trial or within 12 months prior to randomisation.

### Outcomes

The co-primary outcomes in this trial are cumulative pain, measured daily using a 0–10 VAS for the first 3 days following surgery, and the QoR-40 at 72 h. The QoR-40 is a global measure of quality of recovery. It incorporates five dimensions of health: patient support, comfort, emotions, physical independence and pain; each item is graded on a 5-point Likert scale. QoR-40 scores range from 40 (extremely poor quality of recovery) to 200 (excellent quality of recovery). Pain will be measured using a 0–10 VAS [17] on the evening of surgery and once daily on the following 3 days. Where possible, these assessments will be made at the same time each day, though this time may depend on when the participant receives his/her surgery. Cumulative pain up to 3 days post-surgery will be calculated as the area under the curve for each participant using the summary measures approach [18].

The secondary outcome measures in this trial are as follows:

- Cumulative opioid consumption over 72 h after surgery: all opioids used on evening of surgery (day 0) and on days 1, 2 and 3 will be recorded and a running total of cumulative opioid use calculated.
- Fitness for discharge will be assessed against pre-defined criteria and collected daily until discharge. Patients will be considered fit for discharge when they meet four criteria: (1) ability to mobilise independently; (2) pain score less than or equal to 3 cm on a 10 cm VAS; (3) ability to perform straight leg raise; (4) ability to bend knee to 90 degrees.
- Oxford Knee Score (OKS): a 12-item patient-reported outcome measure designed to measure pain and function after knee replacement surgery [19]. Each question is scored from 0 (“none”, meaning no pain, usually in knee) to 4 (“severe” pain, usually in knee). A total score is obtained by summing across all 12 items to give a single score with a range from 0 (best score) to 48 (worst score).
- American Knee Society Score (AKSS): Symptoms, Satisfaction, Expectations, Functional Activities. The “Symptoms” category contains two 10-level scales, ranging from “none to “severe” for patients to rate their pain for walking on level ground and on stairs/inclines. There is an additional question regarding how “normal” the knee feels to the patient. “Patient Satisfaction” is a five-question, 40-point scale, and “Patient Expectations” is a three-question, 15-point scale that is collected pre-operatively (at baseline) and post-operatively (up to 72 h after surgery, 6 weeks after surgery and 6 and 12 months after randomisation). The pre-operative questions reflect patients’ opinions on the extent to which they expect that the operation will improve their knee pain and their ability to perform their activities of daily living and recreational activities. The post-operative questions reflect the extent to which the outcome after the operation has met the patient’s pre-operative expectations with respect to pain and function. The “Functional Score” comprises four subgroups (Walking and Standing, Standard Activities, Advanced Activities and Discretionary Activities). The maximum score is 100 [20].
- Cost utility analysis using patient-reported quality of life as the main outcome, obtained using the EuroQol EQ-5D-5 L questionnaire [21] at baseline, on the evening of surgery (day 0), days 1, 2 and 3 following surgery and at 6 weeks and 6 and 12 months.
- Complications: serious adverse events (SAEs), specifically cardiovascular or wound complications within 30 days of surgery. These will be recorded using the Clavien-Dindo Classification [22].

Data management will be performed via a web-based, bespoke trial database (OpenClinica). OpenClinica is a dedicated and validated clinical trials database designed for electronic data capture.

### Recruitment

All patients listed for knee replacement, i.e. TKR and UKR, for end-stage osteoarthritis will be eligible. We expect to recruit a maximum of 10% of patients listed for UKRs, as this would reflect current practice in the UK. The number of UKRs recruited will not be restricted; however, the proportion of procedures of each type will be reported by treatment group. Patients will be identified at outpatient clinics by participating surgeons and their clinical care team. Potential patients may also be identified by the clinical team from surgical waiting lists. Informed consent will be taken by a medically qualified and suitably experienced investigator. Consent will be verbally re-confirmed on the day of surgery. Due to local patient pathways, informed consent and baseline assessments may be completed at the same hospital visit; however, it may be the case that randomisation is not performed on the same day. Where possible, randomisation will occur on the day of surgery either by a member of the clinical research team or a member of the surgical team. Where this is not possible, randomisation may occur on the days prior to surgery. Patients will be blinded to the treatment allocation. Surgeons and anaesthetists will not be blinded. Un-blinding will be performed on an individual basis as clinical need dictates.

### Interventions

The trial drug is 266 mg liposomal bupivacaine. In the intervention arm, the liposomal bupivacaine is admixed with 100 mg of plain bupivacaine hydrochloride. Plain bupivacaine hydrochloride is included alongside the investigational treatment to ensure pain relief in the immediate post-operative period for these patients. In the control arm, 100 mg bupivacaine hydrochloride is administered alone. In both arms, the volume is expanded to 120 ml with normal saline and administered as a single dose intra-operatively by peri-articular infiltration.

The surgeons will be trained in the administration technique, and a guide to the technique will be provided in each operating theatre. With the exception of the trial drug or active control, the pre-, intra- and post-operative analgesia regimes and management will follow local protocols. Peri-operative pain and rehabilitation pathways are not standardised across study sites. They vary across hospitals and may include a variety of regimens, including scheduled opioids and additional nerve blocks, per provider discretion.

Surgeons will be asked about their compliance with the intervention procedure following treatment of each participant.

### Withdrawals

Each participant has the right to withdraw from any aspect of the trial at any time. For many reasons, patients’ operations may be cancelled, or patients may withdraw themselves from the surgical waiting list. If patients have been randomised and then do not go on to have surgery, reasons for cancellation will be recorded. If patients are willing, they will continue to be followed up. If a participant withdraws from the follow-up regime, data up to the point of withdrawal will be collated and analysed accordingly.

### Safety

#### Reporting of complications or adverse events

Within 30 days of the surgery, adverse events need to be recorded if there is a reasonable possibility they are related to the administration of the investigational medicinal product (IMP), the control drug or the knee replacement surgery. Common examples of such events include nausea, constipation and vomiting. If the event is deemed to be serious (i.e. it meets the SAE criteria), then this will be reported on a SAE form. SAEs must be reported throughout the duration of the study and follow-up period (12 months from randomisation). The sites must notify the study office in Oxford of these events within 24 h following knowledge of the event.

All patient deaths, regardless of cause/relatedness, will be reported on the SAE form.

The Reference Safety Information (RSI) (US prescribing information, found at www.dailymed.nlm.nih.gov) lists all known expected adverse reactions in relation to the administration of the trial drug (liposomal bupivacaine). The Summary of Product Characteristics (SmPC) lists all known adverse drug reactions for the control drug (bupivacaine hydrochloride). The assessment of expectedness will be done centrally, independent of the site Principal Investigator, and will be performed against what is documented in the RSI or SmPC.

### Statistics and analysis

#### Sample size

The trial has been powered for two primary endpoints, QoR-40 at 72 h and cumulative pain score from 6 to 72 h, with adjustment for multiplicity using the Bonferroni method [23]. The trial will be assessed as providing evidence of a difference if either of the two primary endpoints is statistically and clinically significant.

The study requires 480 patients (240 per treatment arm) in order to be 90% powered to detect a 5-point difference in global QoR-40 score between groups at a significance level of 0.025 (two-sided, adjusted for multiplicity) assuming that the standard deviation is 15.5 [24]. To allow for 4% loss to follow-up, this has been inflated to 500 patients (250 per treatment arm).

The study also requires a minimum of 225 patients per treatment arm in order to be 90% powered to detect a standardised difference of 33% between groups in cumulative pain score calculated as area under the curve from 6 to 72 h post-surgery at a significance level of 0.025 (two-sided, adjusted for multiplicity). Inflating the sample size to 500 patients (250 per treatment arm) will allow for 10% loss to follow-up on this variable.

Therefore, a total of 500 patients will be randomised (250 per arm).

#### Statistical analysis

The primary objective of improved patient recovery will be assessed by analysing the two primary endpoints: global QoR-40 score at 72 h and cumulative pain score from 6 to 72 h following surgery. The null hypothesis that there is no difference between the treatment arms will be rejected if either of these dual primary outcomes is statistically different at the two-sided 2.5% significance level.

Both of the primary outcomes will be analysed using multi-variate linear regression adjusting for stratification factors and other important prognostic variables, including baseline scores in the case of the QoR-40. The adjusted mean difference between the two groups together with 97.5% confidence intervals will be reported for each of the primary outcomes. If either of the primary outcomes is not normally distributed, an appropriate transformation to normality will be the first approach. The transformation selected will depend on the departure from normality observed. If this is not possible, then non-parametric techniques without adjustment will be used.

As secondary analyses, both pain and QoR-40 measured at 6, 24, 48 and 72 h will be assessed using longitudinal methods to take account of the multiple time points and the correlation between them. Multi-level, multi-variate linear regression modelling with adjustment for the same factors as the primary analysis will be used. Other continuous variables will be analysed using a similar methodology. For binary variables, the number and percentage of patients in each category will be reported for each treatment group and overall. Chi-squared tests will be utilised for comparing the treatments, with additional analyses being undertaken using multi-variable logistic regression with adjustment for stratification and other important prognostic factors if sufficient events have been observed.

For the dual primary outcomes, a significance level of 0.025 (2.5%) will be used and 97.5% confidence intervals will be presented. All secondary analyses will be considered as supporting the primary analyses, and thus, a significance level of 0.05 (5%) will be used and 95% confidence intervals will be presented.

All principal analyses will be undertaken on an intent-to-treat basis; that is, patients will be analysed as they are randomised, with sensitivity analyses being undertaken on the per-protocol population. The principal analyses will be performed for the available case dataset. For each outcome variable, patterns of missing data will be explored, and if there is a substantial amount of missing data on either of the dual primary outcomes, a sensitivity analysis using multiple imputation will explore the impact of missing data on the results.

### Health economic analysis

A cost utility analysis will be performed using patient-reported quality of life, obtained using the EuroQol EQ-5D-5 L questionnaire at baseline, on the evening of surgery (day 0), on days 1, 2 and 3 following surgery and at 6 weeks, 6 months and 1 year. Following National Institute for Health and Care Excellence (NICE) recommendations [25], the EQ-5D-5 L responses will be mapped onto the EQ-5D-3 L valuation set to derive the utility values [26]. Quality-adjusted life years (QALYs) will be calculated using the area under the curve approach, which involves estimating the average EQ-5D utility between each follow-up time weighted by survival time.

To estimate healthcare costs associated with knee replacement and trial drug, information will be collected for each patient in the trial on the resources used during the initial surgery (including time in theatre, type of procedure, analgesic medication, complications and length of stay) and on subsequent related healthcare use (e.g. primary care visits, outpatient visits, hospital re-admissions, etc.) over the first year following randomisation. Information will also be collected on out-of-pocket expenses (e.g. over-the-counter medications, equipment, travel costs to attend consultations, private practitioners, etc.), impact on informal carers and loss of earnings resulting from knee replacement.

The best practice methods will be followed to address missing data in cost-effectiveness studies [27]. Descriptive statistics (means, standard deviations) will be provided for resource use, costs and EQ-5D utilities in each trial arm and at each follow-up time point. If appropriate, we will estimate the incremental cost-effectiveness ratio (ICER) by dividing the mean cost difference between trial arms by the mean QALY difference. Differences between trial arms will be estimated using linear regression controlling for treatment allocation and the same factors as used for the primary endpoints. Uncertainty around the ICER will be determined using non-parametric bootstrapping and presented graphically in a cost-effectiveness scatter plot and cost-effectiveness acceptability curves [28].

### Trial committees

A Data and Safety Monitoring Committee (DSMC) will be appointed to safeguard the interests of the trial participants, to assess the safety and efficacy of the interventions during the trial and to monitor the overall conduct of the trial, protecting its validity and credibility. The DSMC will not be asked to perform any formal interim analyses of effectiveness. The DSMC will be independent of the trial investigators and sponsor and will adopt a DAMOCLES charter that defines its terms of reference and operation in relation to oversight of the trial. The DSMC may advise the chair of the Trial Steering Committee (TSC) at any time if, in its view, the trial should be stopped for ethical reasons. The DSMC will comprise two independent medically qualified clinicians and a statistician.

A TSC will also be appointed whose primary function is to act as an oversight body for the trial on behalf of the sponsor and funding body. The TSC will be chaired by an independent member and act as appropriate on the recommendations of the DSMC. The TSC ultimately carries responsibility for deciding if the trial needs to be stopped on the grounds of safety.

### Dissemination

The trial results will be disseminated, regardless of the magnitude or direction of effect, to key stakeholders and patients in several ways: peer-reviewed, open-access journals; funder reports; presentations at key scientific meetings, made available on specialist websites; feedback to trial participants; press releases at collaborating institutions; and cooperation with the patient representatives to disseminate to the wider public.

The criteria for authorship will be taken from the International Committee of Medical Journal Editors. Those who do not meet the authorship criteria but contributed to aspects of the study design or drafting of work will be acknowledged as contributors. Those who were solely involved in trial conduct (e.g. staff at recruiting sites) will be acknowledged as collaborators.

## Discussion

This research will further knowledge of the use of opioid-sparing techniques in the management of patients undergoing knee replacement surgery. Clinical and cost effectiveness of the liposomal bupivacaine intervention will be considered. Results may influence post-operative patient management regimes.

### Trial status

The first patient was randomised to the trial in March 2018. Recruitment for the study is ongoing and is expected to finish in February 2020. This paper is based on the latest version of the protocol, v5 dated November 2018.

## Supplementary information

**Additional file 1.** SPIRIT 2013 checklist: recommended items to address in a clinical trial protocol and related documents*.

## Data Availability

Not applicable.

## Abbreviations

AKSS: American Knee Society Score
ASA: American Society of Anesthesiologists
KR: Knee replacement
NHS: National Health Service
OCTRU: Oxford Clinical Trials Research Unit
OKS: Oxford Knee Score
QoR-40: Quality of Recovery 40
RCT: Randomised controlled trial
RSI: Reference Safety Information
SAE: Serious adverse event
SmPC: Summary of Product Characteristics
SPIRIT: Standard Protocol Items: Recommendations for Interventional Trials
TKR: Total knee replacement
UKR: Unicompartmental knee replacement
